# Anfragen zu und Praxis von Suizidassistenz – Ergebnisse einer Befragung unter Mitgliedern der Deutschen Gesellschaft für Palliativmedizin (DGP)

**DOI:** 10.1007/s00103-025-04087-5

**Published:** 2025-06-26

**Authors:** Julian Turiaux, Isabel Sophie Burner-Fritsch, Martin Fegg, Georg Marckmann, Claudia Bausewein

**Affiliations:** 1 Klinik und Poliklinik für Palliativmedizin, LMU Klinikum München, Marchioninistr. 15, 81377 München, Deutschland; 2 Gemeinschaftspraxis für Psychotherapie Prof. Dr. Fegg & Kolleg:innen, Sonnenstr. 10, 80331 München, Deutschland; 3 Institut für Ethik, Geschichte und Theorie der Medizin, Ludwig-Maximilians-Universität München, Lessingstr. 2, 80336 München, Deutschland

**Keywords:** Suizidassistenz, Freiverantwortlichkeit, Todeswunsch, Deutsche Gesellschaft für Palliativmedizin, § 217 StGB, Assisted suicide, Free responsibility, Desire to die, German Association for Palliative Medicine, § 217 German Criminal Code

## Abstract

**Hintergrund:**

Es liegen kaum Daten zu Anfragen zu und Praxis von Suizidassistenz aus Deutschland vor. Ziel der Studie ist die Beschreibung von Suizidassistenzanfragen im Kontext der deutschen Palliativversorgung, des Umgangs damit sowie der Rahmenbedingungen von Suizidassistenzen.

**Methode:**

Online-Umfrage (10/23–01/24) unter allen Mitgliedern der Deutschen Gesellschaft für Palliativmedizin (*n* = 5667), Fragebogenentwicklung auf Basis einer Literaturrecherche und Expert:innen-Pilotierung, quantitativ-deskriptive Auswertung mit IBM SPSS Statistics©, qualitativ-inhaltsanalytische Auswertung mit MAXQDA©.

**Ergebnisse:**

Aufrufrate 434/2585 (16,8 %), Teilnehmendenrate 337/434 (77,6 %), Abschlussrate 272/337 (80,7 %), Analyse von *n* = 321 Datensätzen. 58,9 % (189/321) haben seit 2020 konkrete Suizidassistenzanfragen erhalten. In 93,8 % (210/224) der Fälle lag eine Erkrankung vor, davon in 52,9 % (111/210) Krebs. Häufigster Grund für Suizidwunsch: Autonomieverlust (153/224, 68,3 %). Eher/überhaupt keine Zustimmung der Teilnehmenden zu folgenden Aussagen: in 23,6 % (52/220) der Fälle, dass die suizidbegehrende Person über existierende Hilfemöglichkeiten informiert war, in 26,8 % (59/220) der Fälle, dass der Suizidwunsch von Dauerhaftigkeit und innerer Festigkeit geprägt war. Strukturierte Erfassung von Suizidwünschen in 52,5 % (114/217) der Fälle. Bei 4/10 Suizidassistenzen lagen Begutachtung und Assistenz in einer Hand.

**Diskussion:**

Die Mehrheit der Teilnehmenden erhielt seit 2020 konkrete Suizidassistenzanfragen. In vielerlei Hinsicht wurde kompetent darauf reagiert. In einigen Fällen wurde eine eingeschränkte Freiverantwortlichkeit festgestellt. Eine strukturierte Erfassung von Suizidwünschen sollte ausnahmslos erfolgen. Standards und Schulungen sind notwendig, um einen professionellen Umgang mit Suizidassistenzanfragen gewährleisten zu können.

## Abstract (en)

**Background:**

There are hardly any data available on requests for and performance of assisted suicide in Germany. This study aims to describe requests for assisted suicide in the context of German palliative care, how the requests are handled, and under which conditions assisted suicide is performed.

**Method:**

Online survey (October 2023 to January 2024) among all members of the German Association for Palliative Medicine (*n* = 5667). Questionnaire development based on literature research and piloting with experts. Quantitative data analysis with IBM SPSS Statistics©. Qualitative data analysis with MAXQDA©.

**Results:**

The view rate was 434/2585 (16.8%), the participation rate was 337/434 (77.6%), and the completion rate was 272/337 (80.7%); *n* = 321 datasets were analyzed. Of the participants, 58.9% (189/321) have received specific requests for assisted suicide since 2020. In 93.8% (210/224), the suicidal person suffered from an illness, predominantly cancer (111/210; 52.9%). The most common reason for a suicide wish was loss of autonomy (153/224; 68.3%). Participants tended not to or did not agree at all with the following statements: in 23.6% (52/220), the suicidal person was informed about support options; in 26.8% (59/220), the suicide wish was firm and deliberate. Structured assessment of suicide wishes was performed in 52.5% (114/217). Assessment of free responsibility and assistance was carried out by the same person in 4/10 cases of assisted suicide.

**Conclusion:**

Most participants have received specific requests for assisted suicide since 2020. In many respects, participants dealt with requests competently. In some cases, participants found that free responsibility was impaired. Suicide wishes should be assessed in a structured way without exception. Standards and training are necessary to ensure that requests for assisted suicide are handled professionally.

## Hintergrund

In seinem wegweisenden Urteil erklärte das Bundesverfassungsgericht (BVerfG) am 26.02.2020 das seit 2015 bestehende Verbot der geschäftsmäßigen Förderung der Selbsttötung (§ 217 StGB) für verfassungswidrig [1]. Es begründete sein Urteil damit, dass der § 217 (StGB) das Recht auf selbstbestimmtes Sterben faktisch entleere. Das BVerfG stellte zudem fest, dass eben jenes Recht nicht auf fremddefinierte Situationen (z. B. bestimmte Lebens- und Krankheitsphasen) beschränkt sei. Es müsse jedoch sichergestellt werden, dass die sog. Freiverantwortlichkeit [2–4] der Entscheidung zur Suizidassistenz gegeben sei, für deren Vorliegen das Gericht notwendige Voraussetzungen formulierte [1]. Nachdem 2 Gesetzesentwürfe in einer Bundestagsabstimmung am 06.07.2023 die erforderliche Mehrheit verfehlten, besteht mehr als 5 Jahre nach dem BVerfG-Urteil keine gesetzliche Neuregelung der Suizidassistenz in Deutschland [5].

Wissenschaftliche Daten zur Häufigkeit von Suizidassistenz bzw. „Tötung auf Verlangen“^1^, zu den Charakteristika von Menschen, die Suizidassistenz/Tötung auf Verlangen in Anspruch nehmen, sowie zu den Rahmenbedingungen von Suizidassistenzen/Tötungen auf Verlangen stammen vorwiegend aus Staaten mit entsprechenden gesetzlichen Regelungen [7–14].

Die aktuelle Datenlage zur Suizidassistenz in Deutschland beschränkt sich auf Veröffentlichungen von Sterbehilfeorganisationen [15, 16], mehrere Publikationen von Gleich et al., die Suizidassistenzfälle in München im Zeitraum von 2020–2023 untersuchten [17–21], und auf Umfragen innerhalb medizinischer Fachgesellschaften [22–27].

Aus einer Umfrage von 2016 ist bekannt, dass Professionelle in der Palliativversorgung häufig mit Suizidassistenzanfragen konfrontiert werden und in seltenen Fällen auch Suizidassistenz durchführen [22]. Mit dem vergleichsweise liberalen BVerfG-Urteil könnte sich die Situation in Deutschland noch einmal verändert haben. Differenzierte Daten dazu liegen jedoch nicht vor. Primäres Ziel der Studie ist die Beschreibung der gegenwärtigen Situation in Bezug auf Suizidassistenzanfragen und -praxis innerhalb der deutschen Palliativversorgung. Sekundäre Ziele sind die detaillierte Beschreibung der Suizidassistenzanfragen, des Umgangs damit sowie der Rahmenbedingungen durchgeführter Suizidassistenzen.

## Methode

### Studiendesign und Studienpopulation

Es wurde eine webbasierte Umfrage unter Mitgliedern der Deutschen Gesellschaft für Palliativmedizin (DGP)^2^ mit bekannten E‑Mail-Adressen durchgeführt. Die Durchführung und Berichterstattung erfolgte unter Berücksichtigung der „Checklist for Reporting Results of Internet E‑Surveys“ (CHERRIES; [28]).

### Fragebogen

Der Fragebogen wurde auf Basis einer Literaturrecherche entwickelt und orientiert sich an systematischen Erfassungen von Fällen von Suizidassistenz/Tötung auf Verlangen im Ausland [12–14] und dem „Assisted Suicide Critical Incident Reporting System“ (ASCIRS^3^; [29]). Es erfolgte eine Pilotierung mit 5 Mitgliedern aus dem DGP-Vorstand sowie mit 2 mit dem Nationalen Suizidpräventionsprogramm (NaSPro)^4^ assoziierten Expert:innen. Die finale Version umfasste 45 Fragen zu folgenden 8 Teilbereichen:

1. soziodemografische Daten der Teilnehmenden (6 Fragen),
2. Anfragesituation (2 Fragen),
3. soziodemografische Daten der suizidbegehrenden Personen (8 Fragen),
4. anamnestische Daten der suizidbegehrenden Personen (8 Fragen),
5. Einschätzung der Freiverantwortlichkeitskriterien zum Zeitpunkt der Anfrage (6 Fragen),
6. Umgang mit den Suizidassistenzanfragen (4 Fragen),
7. Rahmenbedingungen durchgeführter Suizidassistenzen (8 Fragen),
8. Häufigkeit der Durchführung von Suizidassistenz und Tötung auf Verlangen (3 Fragen).

Filterfragen reduzierten die Fragenanzahl individuell. Die Teilnehmenden konnten bis zu 5 Suizidassistenzanfragen berichten. Entsprechend häufig wiederholten sich die Fragen der Teilbereiche 3–7.

Die Teilbereiche 1–4, 6 und 7 wurden fast ausschließlich mittels Multiple- und Single-Choice-Fragen operationalisiert. Die Gründe für den Suizidwunsch wurden sowohl mit einer Multiple-Choice-Frage als auch mit einer Freitextfrage erhoben, um zu diesem relevanten Aspekt tiefergehende Informationen zu erhalten. Die Fragen aus Teilbereich 8 erforderten numerische Angaben. Bei einigen Multiple- und Single-Choice-Fragen wurde die Kategorie *Sonstiges* gemeinsam mit einem Freitextfeld zur Verfügung gestellt. Zudem bestand bei allen Fragen die Möglichkeit, keine Angabe zu machen (*keine Angabe, weiß nicht*)*.* Die Fragen zu Teilbereich 5 wurden mithilfe einer 4‑stufigen Likert-Skala operationalisiert (*stimme voll und ganz zu, stimme eher zu, stimme eher nicht zu, stimme gar nicht zu*). Zudem bestand die Antwortoption *weiß nicht*. Teilbereich 5 beinhaltete zudem eine Frage nach der Urteilssicherheit bei der Einschätzung der Freiverantwortlichkeitskriterien, die ebenso mit einer 4‑stufigen Likert-Skala operationalisiert wurde (*sicher, eher sicher, eher unsicher, unsicher*).

Die auf Basis der Pilotierung geschätzte Bearbeitungszeit betrug ca. 5–30 min. Mittels eines Zurück-Buttons konnten die Teilnehmenden Antworten auf vorherigen Seiten korrigieren.

### Durchführung der Befragung

Die Umfrage wurde mit LimeSurvey (https://www.limesurvey.org/de/) programmiert. Die Verwendung von Cookies sollte Mehrfachteilnahmen verhindern.

Die Umfrage wurde im regulären Newsletter der DGP angekündigt und der Umfragelink mit weiteren Informationen zur Studie an die DPG-Mitglieder verschickt. Es handelte sich um eine geschlossene Umfrage, da nur die kontaktierten Personen Zugang zu der Umfrage hatten. Durch einen Klick auf den Umfragelink gelangten die Teilnehmenden zur Startseite von LimeSurvey. Hier erhielten sie weiterführende Informationen zum Hintergrund und Ziel der Umfrage sowie Hinweise zur Bearbeitung und zum Datenschutz. Eine datenschutzrechtliche Einwilligungserklärung war notwendig, um teilzunehmen. Jeweils nach 4 und 8 Wochen wurden die DGP-Mitglieder im Rahmen des monatlichen Newsletters an die Umfrage erinnert und der Umfragelink erneut zur Verfügung gestellt. Die Datenerhebung erfolgte zwischen dem 26.10.2023 und dem 09.01.2024. Es wurden keine Anreize für die Teilnahme angeboten.

### Datenauswertung

Die Auswertung der Multiple- und Single-Choice-Fragen erfolgte quantitativ-deskriptiv mit der Software IBM SPSS Statistics© (Version 29). Die Antworten wurden anhand absoluter und relativer Häufigkeiten ausgewertet. Da auch unvollständig ausgefüllte Fragebögen bei der Auswertung berücksichtigt wurden, sind die berichteten Fallzahlen je nach Variable unterschiedlich hoch. Die Angaben in den Freitextfeldern wurden mittels MAXQDA© qualitativ-inhaltsanalytisch nach Mayring ausgewertet [30]. Die unter „Sonstiges“ gegebenen Antworten wurden thematisch kategorisiert und in absoluten Häufigkeiten erfasst. Die Antworten auf die Freitextfrage zu den Gründen für die Suizidwünsche wurden zunächst deduktiv mit den in der vorangehenden Multiple-Choice-Frage vorgegebenen Antwortkategorien kodiert. Anschließend wurden für damit nicht kodierbare Sequenzen induktiv zusätzliche Kategorien gebildet.

## Ergebnisse

### Antwortraten.

Aufgrund kontinuierlich steigender Mitgliederzahlen wurden die 3 Newsletter an unterschiedlich viele DGP-Mitglieder versandt (5603; 5620; 5667). Die Newsletter wurden auch unterschiedlich häufig geöffnet (2562; 2520; 2674; Mittelwert = 2585). Insgesamt wurde der Umfragelink von 434 Mitgliedern angeklickt. Davon haben 337 Mitglieder die Umfrage begonnen und 272 Mitglieder diese vollständig ausgefüllt. Es wurden insgesamt 321 Datensätze ausgewertet (Einschlusskriterium: mind. Frage nach Suizidassistenzanfragen seit 2020 beantwortet). Daraus ergaben sich folgende Antwortraten: Aufrufrate: 16,8 % (434/2585), Teilnehmendenrate: 77,6 % (337/434), Abschlussrate 80,7 % (272/337).

### Soziodemografische Daten der teilnehmenden DGP-Mitglieder.

Mehr als 60 % der Teilnehmenden waren mind. 51 Jahre alt. Fast 2 Drittel waren weiblich und knapp 60 % der Sektion „Ärztinnen und Ärzte“ zugehörig. Etwas weniger als die Hälfte war in einem SAPV-Team (spezialisierte ambulante Palliativversorgung) oder auf einer Palliativstation tätig. Weitere Angaben in Tab. 1.

**Tab. 1:** Teilnehmende DGP-Mitglieder: soziodemografische Daten und Häufigkeit von Suizidassistenzanfragen/-praxis

	Absolute Häufigkeit (*n*)	Relative Häufigkeit (%)
**Soziodemografische Daten**		
*Alter*		
< 30 Jahre	4	1,2
30–40 Jahre	40	12,5
41–50 Jahre	71	22,1
51–60 Jahre	115	35,8
61–70 Jahre	70	21,8
> 70 Jahre	17	5,3
Keine Angabe	4	1,2
*Geschlecht*		
Weiblich	207	64,5
Männlich	109	34,0
Divers	1	0,3
Keine Angabe	4	1,2
*DGP-Sektion*		
Ärztinnen und Ärzte	191	59,5
Pflege	79	24,6
Soziale Arbeit	10	3,1
Psychologie	6	1,9
Seelsorge/Spiritual Care	10	3,1
Physiotherapie-Ergotherapie-Logopädie	3	0,9
Pharmazie	1	0,3
Geistes- und sozialwissenschaftliche Berufe	9	2,8
Künstlerische Therapien	1	0,3
Keine Angabe	11	3,4
*Einrichtung (vorwiegende Tätigkeit)*		
Praxis	37	11,5
Palliativstation	58	18,1
Andere Station im Krankenhaus	28	8,7
Palliativdienst im Krankenhaus	22	6,9
SAPV-Team	91	28,3
Stationäres Hospiz	10	3,1
Ambulanter Hospizdienst	20	6,2
Tagesklinik	2	0,6
Außerhalb der Patient:innenversorgung	29	9,0
Keine Angabe	6	1,9
Sonstiges	18	5,6
*Berufserfahrung allgemein*		
< 5 Jahre	6	1,9
5–10 Jahre	29	9,0
11–15 Jahre	31	9,7
16–20 Jahre	32	9,9
> 20 Jahre	220	68,5
Keine Angabe	3	0,9
*Berufserfahrung spezialisierte Palliativversorgung*		
Keine	31	9,7
< 5 Jahre	54	16,8
5–10 Jahre	89	27,7
11–15 Jahre	77	24,0
16–20 Jahre	34	10,6
> 20 Jahre	28	8,7
Keine Angabe	8	2,5
**Häufigkeit von Suizidassistenzanfragen/-praxis**		
*Suizidassistenzanfragen seit 2020*		
Ja	189	58,9
Nein	126	39,3
Keine Angabe	6	1,9
*Anzahl an Suizidassistenzanfragen seit 2020*		
1–3	95	50,3
4–6	57	30,2
7–9	11	5,8
10–12	13	6,9
> 12	12	6,3
Keine Angabe	1	0,5
*Anzahl durchgeführter Suizidassistenzen seit 2020*		
0	117	79,1
1	8	5,4
3	1	0,7
4	1	0,7
5	1	0,7
Keine Angabe	20	13,5
*Anzahl durchgeführter Suizidassistenzen vor 2020*		
0	221	80,4
1	5	1,8
2	2	0,7
4	1	0,4
Keine Angabe	46	16,7
*Anzahl durchgeführter Tötungen auf Verlangen*		
0	231	84,0
1	1	0,4
2	1	0,4
Keine Angabe	42	15,3

Gesamtsummen > 100 % ergeben sich durch Rundung. Unterschiedliche Fallzahlen je Variable resultieren aus der Einbeziehung unvollständig ausgefüllter Fragebögen.

*DGP* Deutsche Gesellschaft für Palliativmedizin, *SAPV* spezialisierte ambulante Palliativversorgung

### Häufigkeit von Suizidassistenzanfragen und -praxis.

Knapp 60 % der Teilnehmenden haben seit 2020 konkrete Suizidassistenzanfragen erhalten. Etwas mehr als 7 % gaben an, seit 2020 Suizidassistenz geleistet zu haben. Weitere Angaben in Tab. 1.

### Soziodemografische und anamnestische Daten der suizidbegehrenden Personen.

Knapp 70 % der suizidbegehrenden Personen waren mind. 61 Jahre alt. Bei 93,8 % der Suizidassistenzanfragen litt die suizidbegehrende Person an einer Erkrankung (> 50 % Krebs). Knapp 8,5 % haben bereits einen Suizidversuch unternommen. Weitere Angaben in Tab. 2.

**Tab. 2:** Suizidbegehrende Personen: soziodemografische und anamnestische Daten

	Absolute Häufigkeit (*n*)	Relative Häufigkeit (%)
**Soziodemografische Daten**		
*Alter*		
< 20 Jahre	2	0,9
20–30 Jahre	1	0,4
31–40 Jahre	6	2,6
41–50 Jahre	14	6,0
51–60 Jahre	40	17,1
61–70 Jahre	54	23,1
71–80 Jahre	63	26,9
81–90 Jahre	37	15,8
> 90 Jahre	9	3,8
Keine Angabe	8	3,4
*Geschlecht*		
Weiblich	114	48,7
Männlich	109	46,6
Divers	11	4,7
*Partnerschaft*		
Ja	127	55,2
Nein	86	37,4
Nicht bekannt	10	4,3
Keine Angabe	7	3,0
*Wohnsituation*		
Allein zu Hause lebend	61	26,6
Mit An‑/Zugehörigen im Haushalt lebend	132	57,6
In Pflege- oder Altenheim lebend	19	8,3
In stationärem Hospiz lebend	5	2,2
Nicht bekannt	1	0,4
Keine Angabe	6	2,6
Sonstiges	5	2,2
*Höchster Bildungsabschluss*		
Ohne Schulabschluss	5	2,2
Hauptschulabschluss	6	2,6
Realschulabschluss	20	8,7
Allgemeine/Fachhochschulreife	32	14,0
Hochschulabschluss	65	28,4
Nicht bekannt	82	35,8
Keine Angabe	19	8,3
Nationalität*		
Deutsch	221	96,1
Sonstiges	9	3,9
**Anamnestische Daten**		
*Erkrankung vorliegend*		
Ja	210	93,8
Nein	13	5,8
Nicht bekannt	1	0,4
Art der Erkrankung*		
Krebserkrankung	111	52,9
Amyotrophe Lateralsklerose	31	14,8
Multiple Sklerose	7	3,3
Demenz	3	1,4
Andere Erkrankung des Nervensystems	21	10,0
Kardiovaskuläre Erkrankung	23	11,0
Atemwegserkrankung	20	9,5
Stoffwechselerkrankung	6	2,9
Gastrointestinale Erkrankung	1	0,5
Erkrankung des Bewegungsapparates	15	7,1
Psychische Erkrankung	15	7,1
Sonstiges	9	4,3
Keine Angabe	2	1,0
*Suizidwunsch besteht seit*		
< 1 Woche	13	5,9
1–3 Wochen	29	13,1
1–3 Monate	56	25,3
4–6 Monate	30	13,6
7–9 Monate	4	1,8
10–12 Monate	12	5,4
> 12 Monate	36	16,3
Nicht bekannt	38	17,2
Keine Angabe	3	1,4
*Suizidwunsch mit An‑/Zugehörigen kommuniziert*		
Ja	163	73,8
Nein	33	14,9
Nicht bekannt	23	10,4
Keine Angabe	2	0,9
*Suizidversuche in der Vorgeschichte*		
Ja	19	8,6
Nein	176	79,6
Nicht bekannt	26	11,8
*Anzahl an Suizidversuchen in der Vorgeschichte*		
1	11	57,9
2–4	5	26,3
Nicht bekannt	3	15,8
*Anfragensteller:in*		
Die suizidbegehrende Person selbst	205	87,6
An‑/Zugehörige der suizidbegehrenden Person	27	11,5
Sonstiges	–	
Außerklinisches Ethikkomitee	1	0,4
Pflegeheimpersonal	1	0,4
*In Betreuung bei der angefragten Person*		
Ja	174	74,7
Nein	59	25,3

Gesamtsummen > 100 % ergeben sich durch Rundung. Unterschiedliche Fallzahlen je Variable resultieren aus der Einbeziehung unvollständig ausgefüllter Fragebögen.

* Mehrfachantworten möglich

### Gründe für Suizidwünsche.

**Abb. 1:**
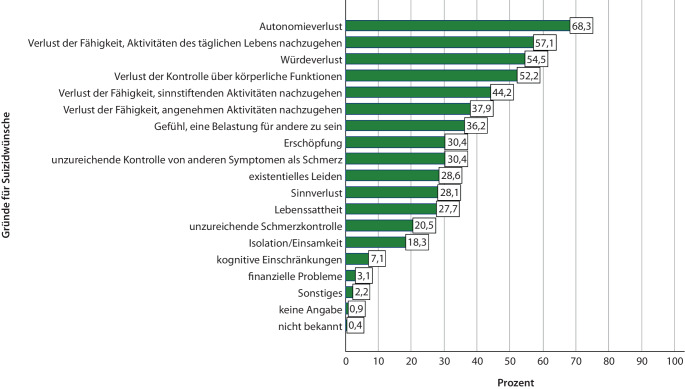
Gründe für Suizidwünsche (*n* = 224; Mehrfachantworten möglich; angegebene Gründe nicht unabhängig voneinander). (Quelle: eigene Abbildung)

Bei der geschlossenen Frage zu den Gründen für die Suizidwünsche wurde Autonomieverlust mit ca. 68 % am häufigsten angegeben, gefolgt von Verlust der Fähigkeit, Aktivitäten des täglichen Lebens nachzugehen, Würdeverlust und Verlust der Kontrolle über körperliche Funktionen (jeweils > 50 %). Weitere Angaben in Abb. 1.

Bei der Freitextfrage zu den Gründen für die Suizidwünsche fanden sich am häufigsten das Nichterlebenwollen des Krankheitsprogresses (50/224; 22,3 %), Autonomieverlust oder die Angst davor (20,5 %), Lebenssattheit (12,5 %) sowie die unzureichende Kontrolle von Schmerz (8,9 %) und anderen Symptomen (11,2 %). Weitere Gründe waren: Verlust über körperliche Funktionen (7,1 %), Würdeverlust (6,7 %), Isolation/Einsamkeit (6,3 %), Pflegebedürftigkeit oder die Angst davor (6,3 %), nicht weiter definierte Symptomlast (5,4 %), fehlende Lebensqualität (5,4 %) und das Gefühl, für andere eine Belastung zu sein (4,0 %). In der Kategorie „Sonstiges“ (11,6 %) wurden Gründe genannt wie „Trauer nach jüngst erlittenem Verlust der Ehefrau“, „an der Welt verzweifeln, Angst vor der Zukunft“ oder „schafft es nicht allein“.

### Einschätzung der Freiverantwortlichkeitskriterien.

**Abb. 2:**
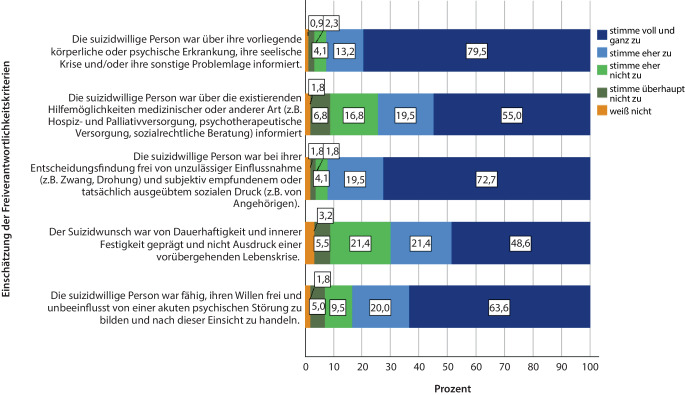
Einschätzung der Freiverantwortlichkeitskriterien (*n* = 220; Gesamtsummen > 100 % ergeben sich durch Rundung). (Quelle: eigene Abbildung)

In jeweils ca. einem Viertel der Fälle stimmten die Teilnehmenden eher nicht oder überhaupt nicht zu, dass die suizidbegehrende Person zum Zeitpunkt der Anfrage über existierende Hilfemöglichkeiten informiert und der Suizidwunsch von Dauerhaftigkeit und innerer Festigkeit geprägt war. In der weiten Mehrheit (192/220; 87,3 %) der Fälle waren sich die Teilnehmenden (eher) sicher bei ihrer Einschätzung. Weitere Angaben in Abb. 2.

### Umgang mit Suizidassistenzanfragen.

**Abb. 3:**
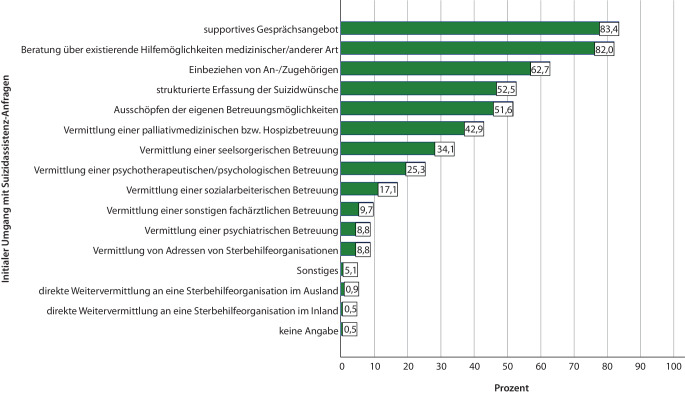
Initialer Umgang mit Suizidassistenzanfragen (*n* = 217; Mehrfachantworten möglich). (Quelle: eigene Abbildung)

In der weiten Mehrheit der Fälle machten die Teilnehmenden zunächst ein supportives Gesprächsangebot und berieten über existierende Hilfemöglichkeiten. In ca. 50 % der Fälle wurden die Suizidwünsche strukturiert erfasst. Weitere Angaben in Abb. 3.

Bei beständigen Suizidwünschen erfolgte in knapp 3 Viertel (84/113; 74,3 %) der Fälle eine weitere Betreuung, ohne Suizidassistenz zu leisten. In 12,4 % der Fälle wurden Adressen von Sterbehilfeorganisationen vermittelt. In knapp jedem 11. berichteten Fall (8,8 %) wurde Suizidassistenz geleistet. In 3,5 % der Fälle wurde die Betreuung beendet. In jeweils 1,8 % der Fälle erfolgte eine direkte Weitervermittlung an eine:n Ärzt:in, der:die zu Suizidassistenz bereit war bzw. an eine Sterbehilfeorganisation im Inland. In 7,1 % wurden Sonstiges-Angaben (z. B. 2‑mal „ethische Fallbesprechung“), in 4,4 % wurde keine Angabe gemacht.

### Gründe dafür, keine Suizidassistenz geleistet zu haben.

In ca. der Hälfte (43/83; 51,8 %) der Fälle, in denen keine Suizidassistenz geleistet wurde, begründeten die Teilnehmenden dies damit, dass sie es ablehnten, selbst Suizidassistenz zu leisten. In 22,9 % der Fälle fühlten sie sich nicht kompetent, Suizidassistenz zu leisten. In 16,9 % der Fälle hatten die Teilnehmenden Sorgen hinsichtlich juristischer Konsequenzen. In jeweils 8,4 % der Fälle wurde angegeben, dass die Voraussetzungen für eine freiverantwortliche Suizidentscheidung nicht erfüllt waren bzw. dass Suizidassistenz eine ärztliche Aufgabe ist. In 7,2 % der Fälle lehnten die Teilnehmenden Suizidassistenz grundsätzlich ab. In ca. einem Viertel der Fälle (26,5 %) wurden sonstige Gründe angegeben, die mehrheitlich organisationsbezogen waren. Hier gaben die Teilnehmenden an, dass im Team ein Beschluss gegen die Durchführung von Suizidassistenz gefasst wurde oder dies seitens der Klinik- und Einrichtungsleitungen oder des Trägers so vorgegeben wurde. In 3,6 % der Fälle wurde keine Angabe gemacht.

### Rahmenbedingungen durchgeführter Suizidassistenzen.

In 6 von 10 Suizidassistenzfällen erfolgte vorab eine Prüfung der Voraussetzungen für eine freiverantwortliche Suizidentscheidung durch eine zusätzliche Fachkraft, mehrheitlich durch eine:n Palliativmediziner:in. Weitere Angaben in Tab. 3.

**Tab. 3:** Rahmenbedingungen durchgeführter Suizidassistenzen

	Absolute Häufigkeit (*n*)	Relative Häufigkeit (%)
**Begutachtung durch zusätzliche Fachkraft**		
Ja	6	60
Nein	4	40
**Qualifikation der begutachtenden Fachkraft**		
Fachärzt:in für Neurologie	1	16,7
Palliativmediziner:in	4	66,7
Andere:r Fachärzt:in	1	16,7
**Ort**		
Zuhause der suizidbegehrenden Person	9	90
Stationäres Hospiz	1	10
Anwesende Personen*		
Angefragte Person selbst	8	80
Weitere:r Vertreter:in der Berufsgruppe der angefragten Person	5	50
Vertreter:in einer anderen Berufsgruppe	3	30
An‑/Zugehörige	8	80
Medikamente für Suizideinleitung*		
Thiopental	2	20
Pentobarbital	1	10
Opioide	5	50
Trizyklische Antidepressiva	1	10
Keine Angabe	1	10
*Sonstiges*	–	
Midazolam	3	30
Diazepam	1	10
**Applikationsform der Medikamente für Suizideinleitung**		
Oral	1	10
Intravenös	4	40
Sonstiges	–	
Subkutan	4	40
Intravenös + perkutan	1	10
Begleitmedikation*		
Benzodiazepine	6	60
Antiemetika	1	10
Keine	4	40
Sonstiges (Neuroleptika)	1	10
**Medikation – Einzelfalldarstellung**		
Opioide + Midazolam	2	20
Opioide + Midazolam + Neuroleptika	1	10
Opioide + Diazepam	1	10
Opioide allein	1	10
Thiopental	2	20
Pentobarbital	1	10
Trizyklische Antidepressiva + Benzodiazepine + Antiemetika	1	10
Keine Angabe	1	10
**Zeit zwischen Anfrage und Durchführung der Suizidassistenz**		
< 1 Woche	3	30
1–3 Wochen	1	10
1–3 Monate	4	40
> 3 Monate	2	20

Gesamtsummen > 100 % ergeben sich durch Rundung.

* Mehrfachantworten möglich

### Suizidassistenzfälle.

In der Teilstichprobe der durchgeführten Suizidassistenzen (10/113; 8,8 %) ergaben sich einzelne deskriptive Besonderheiten. Die Suizide vollzogen überwiegend Frauen (7/10), in 2 von 10 Fällen lag keine Erkrankung vor und in 9 von 10 Fällen ergab sich eine volle Zustimmung zu folgenden Freiverantwortlichkeitskriterien: Informiertheit über existierende Hilfemöglichkeiten, Dauerhaftigkeit und innere Festigkeit des Entschlusses.

## Diskussion

Die vorliegende Studie stellt erstmals differenzierte Daten zu Suizidassistenzanfragen und -praxis bei Professionellen in der deutschen Palliativversorgung bereit. Die Ergebnisse beruhen auf einer mehrheitlich weiblichen Stichprobe von vorwiegend Ärzt:innen und Pflegenden, die zumeist in der spezialisierten Palliativversorgung tätig sind und mehrheitlich mehr als 20 Jahre Berufserfahrung aufweisen.

### Häufigkeit von Suizidassistenzanfragen und -praxis

In der vorliegenden Umfrage gaben ca. 60 % der Teilnehmenden an, seit 2020 konkrete Suizidassistenzanfragen erhalten zu haben. Eine ältere sowie eine aktuelle Umfrage zu Einstellungen zu und Erfahrungen mit Suizidassistenz innerhalb der DGP ergaben, dass knapp 75 % der Teilnehmenden um eine eigene Beteiligung bzw. Mithilfe bei einer Suizidassistenz gebeten wurden [22, 26]. In einer Umfrage von Schildmann et al. gaben ca. 60 % der teilnehmenden Mitglieder der Deutschen Gesellschaft für Hämatologie und Medizinische Onkologie (DGHO) an, bereits um Informationen zu Suizidassistenz gebeten worden zu sein [24]. Knapp halb so viele Teilnehmende gaben an, konkrete Bitten erhalten zu haben, ein Suizidmittel zu verschreiben [23].^5^ Ein Vergleich der hier vorgestellten Umfrageergebnisse mit den anderen Studien gestaltet sich aufgrund der unterschiedlichen Formulierungen der Fragen (z. B. *um Mithilfe gebeten *vs. *konkrete Anfragen erhalten*), der unterschiedlichen Stichprobenzusammensetzung (in dieser Umfrage ca. 40 % nichtärztliche Berufsgruppen) sowie der Begrenzung des Zeitraums in dieser Umfrage (*seit 2020*) schwierig. In der Gesamtschau kann jedoch festgehalten werden, dass die Mehrheit der Professionellen, die terminal Erkrankte betreuen, Suizidassistenzanfragen erhält.

In Bezug auf die Häufigkeit von durchgeführten Suizidassistenzen lässt sich ein Trend feststellen. So war der Anteil an Teilnehmenden der vorliegenden Umfrage, die angaben, seit 2020 Suizidassistenz geleistet zu haben, mehr als doppelt so hoch (7,4 %) wie der Anteil an Teilnehmenden früherer Umfragen, die angaben, jemals Suizidassistenz geleistet zu haben [22, 23]. Dies könnte auf die Liberalisierung der Suizidassistenz in Deutschland im Zuge des BVerfG-Urteils zurückzuführen sein. Diese Annahme wird durch den Befund gestützt, dass nur 2,9 % der Teilnehmenden der vorliegenden Studie angaben, vor 2020 Suizidassistenz geleistet zu haben. Auch andere Daten aus Deutschland zeigen einen Anstieg an Suizidassistenzfällen seit dem BVerfG-Urteil [15, 19]. Damit übereinstimmend ist auch im Ausland eine konstante Zunahme an Fällen von Suizidassistenz/Tötung auf Verlangen seit Inkrafttreten der jeweiligen Gesetze zu konstatieren [31].

### Soziodemografische und anamnestische Daten der suizidbegehrenden Personen

In wesentlicher Übereinstimmung mit Daten aus dem Ausland stammte die Mehrheit der Anfragen von hochbetagten Menschen, die hoch gebildet waren, sich in einer Partnerschaft befanden und vorwiegend an Krebs, aber auch an neurologischen oder kardiovaskulären Erkrankungen litten [7–14]. Abweichend von den meisten Ländern [7–9, 11–14], jedoch in Übereinstimmung mit der Schweiz [9, 10], lagen in dieser Studie etwas mehr Anfragen von Frauen als von Männern vor.

Auch in der Studie von Gleich et al., im Rahmen derer vorwiegend durch Sterbehilfeorganisationen vermittelte Suizidassistenzen im Zeitraum 2020–2023 untersucht wurden, war ein Frauenüberhang zu beobachten und handelte es sich vorwiegend um hochbetagte Menschen, die signifikant häufiger als die Vergleichsgruppe (konventionelle Suizide) einen akademischen Beruf ausübten [19]. Unterschiede zeigten sich hinsichtlich der dokumentierten Erkrankungen. Im Kollektiv von Gleich et al. war der Anteil von Krebserkrankungen weniger als halb so groß wie in dieser Studie. Der Anteil von psychischen Erkrankungen (vorwiegend Depression) hingegen erwies sich um ein Vielfaches größer [19]. Dieser Befund deutet daraufhin, dass sich Personen, die bei einer Sterbehilfeorganisation Suizidassistenz suchen, im Mittel von denjenigen unterscheiden, die bei Professionellen in der Palliativversorgung Suizidassistenz anfragen. Dafür spricht auch, dass im Kollektiv von Gleich et al. in keinem Fall eine palliativmedizinische Betreuung dokumentiert war [17].

Wenngleich psychische Erkrankungen einen wichtigen Risikofaktor für Suizid darstellen [32, 33] und sich deren Anteil im Kollektiv von Gleich et al. als wesentlich höher als in der vorliegenden Studie erwies, war der Anteil vorangegangener Suizidversuche in beiden Studien ähnlich hoch [19]. Dies unterstreicht die Bedeutung einer staatlichen Suizidpräventionsstrategie auch für Menschen, die keine psychische Erkrankung aufweisen. Ein entsprechender Gesetzesentwurf wurde im November 2024 vom Bundesministerium für Gesundheit vorgelegt [34].

### Gründe für Suizidbegehren

Wie auch in anderen Studien wurden in der vorliegenden oft mehrere Gründe für die Anfrage nach Suizidassistenz angegeben [35]. Übereinstimmend mit Daten aus dem Ausland befanden sich auch in dieser Studie Autonomieverlust, Würdeverlust sowie der Verlust der Fähigkeit, sinnstiftenden/angenehmen Aktivitäten nachzugehen, unter den häufigsten Gründen für die Suizidwünsche [11–13]. Gleichzeitig lassen sich diesbezüglich teils erhebliche Unterschiede zwischen den vorhandenen (einschließlich der vorliegenden) Studien finden. Beispielsweise wird eine unzureichende Schmerzkontrolle in Kanada knapp 3‑mal so häufig angegeben wie in der vorliegenden Studie [12]. Solche Unterschiede könnten unter anderem darauf zurückzuführen sein, dass je nach Quelle unterschiedliche und unterschiedlich viele Kategorien vorgegeben werden, die sich teils sprachlich unterscheiden und inhaltlich nicht unabhängig voneinander sind. Auch in der vorliegenden Studie unterschieden sich die Ergebnisse der geschlossenen Frage von denjenigen der offenen Frage. Teilweise konnten die in der offenen Frage angegebenen Gründe nicht eindeutig den Antwortkategorien der geschlossenen Frage zugeordnet werden (z. B. *Symptomlast*). Zudem zeigte sich in den Ergebnissen der offenen Frage, dass hinter dem Begehren nach Suizidassistenz neben konkreten Symptombelastungen und Problemen auch die Angst vor potenziell zukünftig eintretenden Umständen stand. Dies unterstreicht die Relevanz fundierter Aufklärung der Anfragenden über palliativmedizinische und weitere Unterstützungsmöglichkeiten im Krankheitsprogress.

### Umgang mit Suizidassistenzanfragen und Einschätzung der Freiverantwortlichkeitskriterien

Gemessen an den Empfehlungen der DGP [36] reagierten die Teilnehmenden in vielerlei Hinsicht kompetent auf Suizidassistenzanfragen. Eine umfassende Aufklärung über Alternativen zum Suizid ist erforderlich, um zu verhindern, dass die suizidbegehrende Person ihren Entschluss auf Basis einer Fehleinschätzung und damit nicht freiverantwortlich trifft [1]. In der vorliegenden Studie haben die Teilnehmenden in den allermeisten Fällen über existierende Hilfemöglichkeiten informiert. Der Stellenwert dieses Vorgehens wird durch die Einschätzung der Teilnehmenden dieser Studie deutlich, dass das Freiverantwortlichkeitskriterium der *Informiertheit über existierende Hilfemöglichkeiten* bei ca. einem Viertel der Suizidassistenzanfragen (eher) nicht erfüllt war. Umso kritischer erscheint das Ergebnis einer Studie von Gleich et al., dass bei Suizidassistenzen unter Beteiligung von Sterbehilfeorganisationen in ca. einem Drittel der Fälle keine Alternativen zum Suizid genannt wurden [21].

Eine Voraussetzung dafür, die Freiverantwortlichkeit beurteilen und ggf. zielgerichtete Hilfsangebote machen zu können, stellt eine strukturierte Erfassung der Suizidwünsche dar [36]. Beispielsweise wäre es für die Beurteilung des Kriteriums der *Dauerhaftigkeit und inneren Festigkeit des Suizidwunsches *unter anderem erforderlich, den Verlauf des Suizidwunsches sowie mögliche innere Ambivalenzen zu erheben [37]. Teilnehmende der vorliegenden Studie nahmen eine strukturierte Erfassung der Suizidwünsche nur in ca. 50 % der Fälle vor. Gleichzeitig kamen diese in mehr als einem Viertel der Fälle zu der Einschätzung, dass das Kriterium der *Dauerhaftigkeit und inneren Festigkeit des Suizidwunsches *zum Zeitpunkt der Anfrage (eher) nicht erfüllt war. Dieser Befund verdeutlicht die Notwendigkeit von Schulungen zum Umgang mit Suizidwünschen für Professionelle in der Palliativversorgung. Entsprechende Angebote existieren bereits [38] und sollten weiter ausgebaut werden.

In manchen Fällen von Suizidassistenzanfragen vermittelten die Teilnehmenden Adressen von Sterbehilfeorganisationen, in einzelnen Fällen erfolgte sogar eine direkte Vermittlung an eine solche Organisation. Einzelfallentscheidungen, Menschen beim Suizid zu unterstützen, fallen in den Gewissensbereich und sind zu respektieren [36]. Der Einbezug von Sterbehilfeorganisationen sollte aus Sicht der Autor:innen insofern kritisch reflektiert werden, als Studien von Gleich et al. nahelegen, dass prozedurale Schutzkonzepte bei Suizidassistenzen, die von Sterbehilfeorganisationen vermittelt werden, nur unzureichend eingehalten werden [21]. Klare Richtlinien könnten Mitarbeitende der Palliativversorgung im professionellen Umgang mit Suizidassistenzanfragen unterstützen. Eine entsprechende Leitlinie wurde bereits angemeldet [39].

### Rahmenbedingungen durchgeführter Suizidassistenzen

Korrespondierend mit den Daten aus München [19] und den USA [7] fand die große Mehrheit der berichteten Suizidassistenzen im Zuhause der suizidbegehrenden Personen statt. In der Schweiz dagegen werden Suizidassistenzen am häufigsten in Räumlichkeiten von Sterbehilfeorganisationen durchgeführt [8, 40]. In den Niederlanden und Kanada werden fast ausschließlich Tötungen auf Verlangen durchgeführt [12, 14]. Während diese in den Niederlanden am häufigsten zu Hause stattfinden [14], werden diese in Kanada auch häufig in Kliniken durchgeführt [12].

Übereinstimmend mit den Daten von Gleich et al. waren auch in der vorliegenden Stichprobe in ca. 80 % der durchgeführten Suizidassistenzen An‑/Zugehörige anwesend [20]. An‑/Zugehörige von Menschen, die Suizidassistenz in Anspruch nehmen, scheinen spezifische psychologische Herausforderungen im Rahmen ihres Trauerprozesses zu erleben [41] und ein erhöhtes Risiko für Depressionen und posttraumatische Belastungsstörungen aufzuweisen [42]. Entsprechend sollten diesem Personenkreis psychosoziale Unterstützungsangebote gemacht werden.

In 4 von 10 Suizidassistenzfällen wurde keine zusätzliche Fachkraft zur Begutachtung der Freiverantwortlichkeit hinzugezogen. Im Kollektiv von Gleich et al. lagen Begutachtung und Suizidassistenz sogar in knapp 78 % der Fälle in einer Hand [21]. Zum Ausschluss von Einschränkungen der Freiverantwortlichkeit sollte eine personelle bzw. organisatorische Trennung der verschiedenen Funktionen bei einer Suizidassistenz gewährleistet werden [3].

Während im Kollektiv von Gleich et al. in ca. 80 % der Fälle Thiopental, in ca. 17 % der Fälle Chloroquin gemeinsam mit Diazepam und Metoclopramid und in ca. 3 % der Fälle Phenobarbital eingesetzt wurde [20], wurde bei den hier berichteten Suizidassistenzen nur in 20 % der Fälle Thiopental und in keinem Fall Chloroquin verwendet. Die am häufigsten eingesetzten Mittel waren dagegen Opioide, meist in Kombination mit Benzodiazepinen.

### Stärken und Limitationen

Eine Stärke dieser Studie ist, dass sich der Fragebogen an systematischen Erfassungen von Suizidassistenzfällen im Ausland orientiert, unter Mitwirkung von Expert:innen aus unterschiedlichen Fachbereichen entwickelt wurde und zahlreiche wesentliche Aspekte von Suizidassistenz beinhaltet. Damit könnte er als Grundlage für die Entwicklung eines nationalen Registers für Suizidassistenz dienen. Im Gegensatz zu systematischen Erfassungen in den USA und Kanada, im Rahmen derer die Gründe für die Suizidwünsche lediglich quantitativ erhoben werden, wurden diese in der vorliegenden Studie zudem qualitativ erfasst.

Beim Vergleich mit Daten zu Fällen durchgeführter Suizidassistenzen im Ausland sollte berücksichtigt werden, dass in dieser Studie allen voran Suizidassistenzanfragen beschrieben werden. Eine Limitation der Studie ist, dass die Rücklaufquote eher gering war (321/5667; 5,7 %) und die Daten damit nicht repräsentativ für die DGP-Mitglieder sind. Dies könnte damit zusammenhängen, dass die DGP-Mitglieder aufgrund der Sensibilität der Thematik sowie Unsicherheit in Bezug auf die Rechtslage möglicherweise gehemmt waren, von eigenen Erfahrungen zu berichten. Zum anderen ist es denkbar, dass sich vorrangig solche Mitglieder von der Umfrage angesprochen gefühlt haben, die bereits in irgendeiner Weise Erfahrungen mit Suizidassistenz gemacht haben. Diesen Aspekten wurde im Vorfeld dadurch begegnet, dass im Anschreiben explizit jedes DGP-Mitglied eingeladen wurde, teilzunehmen, unabhängig von eigenen Erfahrungen mit Suizidassistenz, die Anonymität der Umfrage sowohl in der Einladung als auch auf der Startseite zugesichert wurde und die DGP-Mitglieder 2‑mal an die Umfrage erinnert wurden. Nicht zuletzt sollte berücksichtigt werden, dass die Daten auch nicht repräsentativ für Professionelle in der Palliativversorgung sind. Mehr als 90 % der Teilnehmenden waren in der spezialisierten Palliativversorgung tätig. Damit sind Professionelle in der allgemeinen Palliativversorgung (z. B. Hausärzt:innen, Pflegepersonal in Pflegeheimen) deutlich unterrepräsentiert.

## Fazit

Die Mehrheit der befragten Professionellen in der Palliativversorgung hat seit 2020 Suizidassistenzanfragen erhalten. Die Daten legen nahe, dass Professionelle in der Palliativversorgung seit dem BVerfG-Urteil von 2020 häufiger Suizidassistenzen durchführen. Menschen, die innerhalb der deutschen Palliativversorgung Suizidassistenz anfragen, scheinen in Bezug auf wesentliche soziodemografische und anamnestische Aspekte mit denjenigen übereinzustimmen, die im Ausland Suizidassistenz/Tötung auf Verlangen in Anspruch nehmen. Dagegen scheinen sie wesentlich häufiger an Krebserkrankungen und seltener an psychischen Erkrankungen zu leiden als Menschen, die in Deutschland mithilfe einer Sterbehilfeorganisation assistierten Suizid begehen. Zentrale Freiverantwortlichkeitskriterien waren aus Sicht der Teilnehmenden in bis zu einem Viertel der Fälle zum Zeitpunkt der Anfrage nicht erfüllt. Während das angefragte Personal in vielerlei Hinsicht kompetent auf Suizidassistenzanfragen reagierte, wurden die Suizidwünsche nur in ca. 50 % der Fälle strukturiert erfasst. In 4 von 10 durchgeführten Suizidassistenzen lagen Begutachtung und Durchführung in einer Hand. Diese Ergebnisse unterstreichen die Bedeutung professioneller Standards im Umgang mit Suizidassistenzanfragen und entsprechender Schulungen für Palliativpersonal. Bei der notwendigen Entwicklung einer Registererfassung von Suizidassistenzanfragen und -durchführungen sollte beachtet werden, dass die Gründe für Anfragen in einer Mehrfachauswahl vorgegebener Kategorien mit einem optionalen Freitextfeld angegeben werden.

## Footnotes

1 Anders als in Deutschland ist auch die „Tötung auf Verlangen“, bei der die suizidbegehrende Person die todbringende Handlung nicht selbst vornimmt, in einigen Staaten (z. B. Kanada oder Benelux-Staaten) legal [6].

2 Die DGP ist eine multiprofessionelle Fachgesellschaft mit über 6500 Mitgliedern aus allen Bereichen der Palliativversorgung.

3 ASCIRS ist eine Online-Plattform der Österreichischen Palliativgesellschaft (ÖPG) zur Erfassung von Suizidassistenzfällen.

4 Das NaSPro ist ein bundesweites Netzwerk zur Förderung der Suizidprävention.

5 Bei den beiden zitierten Studien von Schildmann et al. [23, 24] wurde dieselbe Stichprobe untersucht.
